# Hsf transcription factor gene family in peanut (*Arachis hypogaea* L.): genome-wide characterization and expression analysis under drought and salt stresses

**DOI:** 10.3389/fpls.2023.1214732

**Published:** 2023-07-05

**Authors:** Qi Wang, Zhenbiao Zhang, Cun Guo, Xiaobo Zhao, Zhiyuan Li, Yifei Mou, Quanxi Sun, Juan Wang, Cuiling Yuan, Chunjuan Li, Ping Cong, Shihua Shan

**Affiliations:** ^1^ Shandong Peanut Research Institute, Qingdao, China; ^2^ Tobacco Research Institute, Chinese Academy of Agricultural Sciences, Qingdao, China; ^3^ Kunming Branch of Yunnan Provincial Tobacco Company, Kunming, China

**Keywords:** peanut, Hsf, abiotic stress, transcription factor, gene family

## Abstract

Heat shock transcription factors (Hsfs) play important roles in plant developmental regulations and various stress responses. In present study, 46 *Hsf* genes in peanut (*AhHsf*) were identified and analyzed. The 46 *AhHsf* genes were classed into three groups (A, B, and C) and 14 subgroups (A1-A9, B1-B4, and C1) together with their *Arabidopsis* homologs according to phylogenetic analyses, and 46 *AhHsf* genes unequally located on 17 chromosomes. Gene structure and protein motif analysis revealed that members from the same subgroup possessed similar exon/intron and motif organization, further supporting the results of phylogenetic analyses. Gene duplication events were found in peanut *Hsf* gene family via syntenic analysis, which were important in *Hsf* gene family expansion in peanut. The expression of *AhHsf* genes were detected in different tissues using published data, implying that *AhHsf* genes may differ in function. In addition, several *AhHsf* genes (*AhHsf5*, *AhHsf11*, *AhHsf20*, *AhHsf24*, *AhHsf30*, *AhHsf35*) were induced by drought and salt stresses. Furthermore, the stress-induced member AhHsf20 was found to be located in nucleus. Notably, overexpression of *AhHsf20* was able to enhance salt tolerance. These results from this study may provide valuable information for further functional analysis of peanut *Hsf* genes.

## 1 Introduction

The growth and development of plants is significantly constrained by abiotic stresses, such as salt, drought, cold and heat. These abiotic stresses have a negative effect on the quality and yield of crops (Umezawa et al., 2006). In order to cope with environmental stresses, plants form various regulatory mechanisms in the course of long-term evolution (Shinozaki et al., 2003). With the global warming, the effect of heat stress on plants has been paid more and more attention. The heat shock response (HSR), as a universal protection mechanism, was activated when faced with heat stress and other stimulating factors (Schoffl et al., 1998). Meanwhile, the heat shock proteins (HSPs), as molecular chaperone, accumulated rapidly to protect the plant from heat stress by maintaining protein homeostasis and repairing damaged proteins under HSR (Wang et al., 2004; Zhang et al., 2013). Furthermore, expression of *HSP* genes is mainly regulated by the Hsfs on a transcriptional level, which can combine with heat shock elements (HSEs:5’-nGAAnnTTCn-3’) in *HSP* gene promoters (Bienz and Pelham, 1987; Lin et al., 2011). The heat shock transcription factors (Hsfs) are the terminal component of the signal transduction chain, which can mediate the activation of the response to heat stress and/or other stress-stimulated genes (Nover et al., 2001).

Previous studies have reported that the Hsfs have several conserved domains, including DNA binding domain (DBD), oligomerization domain (OD), nuclear localization signal (NLS), nuclear export signal (NES), C-terminal activation domain (CTAD), and repressor domain (RD) (Nover et al., 2001; Kotak et al., 2004; Guo et al., 2008). Among them, DBD is the most conserved domain in Hsf proteins, which is characterized by its hydrophobic core containing a typical helix-turn-helix conserved motif. It is essential for recognizing and binding to the conserved motif of the heat shock element (HSE) in the promoter of the target genes (von Koskull-Doring et al., 2007; Huang et al., 2015). OD (HR-A/B) is composed of two hydrophobic seven peptide repeat regions, and the domain can promote Hsf effectively bind to the HSE in HSP promoter by forming a specific structure (Scharf et al., 2012). CTAD is the least conserved region in Hsf protein sequence, which contains a short peptide AHA motif and is a necessary condition for Hsfs transcriptional activity (Doring et al., 2000). According to the number of amino acid residues connected between HR-A/B, plant Hsf protein families can be divided into three groups: A, B, and C. The number of amino acid residues insertion between HR-A/B is 21 and 7 in group A and group C Hsfs, respectively, whereas group B Hsfs have no amino acid insertion between HR-A/B (Scharf et al., 2012). It is worth noting that only group A Hsfs contain the C-terminal activation motifs (AHA motifs).

To date, a large number of heat shock transcription factors have been identified in different plants, including 21 genes in *Arabidopsis*, 25 in rice (Guo et al., 2008), 27 in potato (Tang et al., 2016), 24 in tomato (Yang et al., 2016), 82 in wheat (Duan et al., 2019), 25 in maize (Lin et al., 2011), and 38 in soybean (Li et al., 2014). In plants, the class A Hsfs consist of majority of the Hsf proteins. Members of class A play important roles in regulating plant response to abiotic stresses (Scharf et al., 2012). In *Arabidopsis*, the expression levels of most heat-stress response genes are regulated by AtHsfA1 (AtHsfA1a, AtHsfA1b, AtHsfA1d and AtHsfA1e). The expression of HS-responsive genes, including transcription factors and molecular chaperones, was significantly decreased in *hsfa1a/b/d* triple mutants. Moreover, the *hsfa1a/b/d/e* quadruple mutant exhibited growth retardation and more sensitive to temperature compared with wide type (Lohmann et al., 2004; Nishizawa-Yokoi et al., 2011; Yoshida et al., 2011). In subgroup A1, AtHsfA1b and AtHsfA1d were reported to enhance drought and heat tolerance, respectively (Bechtold et al., 2013; Higashi et al., 2013). *AtHsfA2* in subgroup A2 and *AtHsfA3* in subgroup A3 have been reported to function in heat tolerance and *AtHsfA2* also enhances anoxia tolerance (Schramm et al., 2006; Charng et al., 2007; Banti et al., 2010; Chen et al., 2010; Friedrich et al., 2021). Meanwhile, overexpression of *AtHsfA3* in *Arabidopsis* increased galactinol level and oxidative stress tolerance (Song et al., 2016). Besides, *AtHsfA6b* in subgroup A6 was highly induced by salt and drought conditions, and positively regulated *Arabidopsis* tolerance to ABA-mediated salt, drought, and heat stresses (Huang et al., 2016).

In addition, Hsf members have been reported to be involved in plant growth and development. For example, in subgroup A9, AtHsfA9 was proved to sever as a seed-specific transcription factor showing to function in regulating expression of heat stress protein-encoding genes during seed development of *Arabidopsis* (Kotak et al., 2007). AtHsfB2a from subgroup B2, was proven to be involved in regulating gametophyte development of *Arabidopsis* (Wunderlich et al., 2014). Besides, *AtHsfB4-*overexpressing transgenic lines displayed short root length phenotype compared with wild type, indicating AtHsfB4 in subgroup B4 was involved in the negative regulation of root development (Begum et al., 2013).

Peanut (*Arachis hypogaea* L.) is an important economic and oil crop in the world, suppling oils and proteins for the human nutrition. The production of peanut was affected by several abiotic stresses, including extreme temperatures, high salinity, and drought, during the growing stage. Utilization of resistant varieties is the most cost-efficient measure to control these stresses. With the availability of the peanut genome database, studies of a number of peanut gene families have been reported, but our understanding of the peanut *Hsf* gene family is very limited. Here, a total of 46 peanut *Hsf* genes were identified from the peanut. Comprehensive analyses on the gene structures, *cis*-acting element composition, chromosome distribution, phylogenetic relationships, and expression patterns were performed. The results of which suggested that the peanut Hsf members might play important roles in peanut development and in response to stresses.

## 2 Materials and methods

### 2.1 Identification and phylogenetic analysis of peanut Hsf proteins

The peanut genome data and the *Arabidopsis* genome data were downloaded from the Peanut Base (https://www.peanutbase.org/) and TAIR (https://www.arabidopsis.org/), respectively. *Arabidopsis* Hsf full-length protein sequences were used as queries to perform BLASTP program against the peanut genome database with an *E*-value of 0.0001. The resulting sequences were then subjected to Pfam (El-Gebali et al., 2019) and SMART (Letunic and Bork, 2018) analyses to detect the presence of the Hsf-type DBD and OD domain. The ProtParam (Wilkins et al., 1999) was used to calculate Mw (molecular weight) and theoretical pI (isoelectric point) of the putative Hsf proteins from peanut.

MAFFT (Katoh et al., 2019) under the default parameters was applied in protein sequences alignment analyses of newly identified peanut Hsf members and previously reported *Arabidopsis* Hsf members. A neighbor-joining (NJ) tree was constructed by MEGA 11 with the following parameters: Poisson correction, 1000 bootstrap values, and pairwise deletion (Tamura et al., 2021).

### 2.2 Gene structure and *cis*-regulatory elements

The structure of peanut *Hsf* genes was analyzed using the gene structure display server (Hu et al., 2015) by comparing the coding sequence (CDS) and genomic sequence obtained from the peanut genome database. The 2000-bp sequences upstream of peanut *Hsf* genes were obtained from peanut genome database as the promoter (Wang et al., 2021). And then the *cis*-regulatory elements of these promoters were identified by PlantCARE (Lescot et al., 2002).

### 2.3 Protein domain and motif analysis

The typical functional structure domains of peanut Hsf protein were analyzed by Pfam and SMART. The nuclear localization signal (NLS) prediction was done by using the online tool (Nguyen Ba et al., 2009). MEME tools (Bailey et al., 2009) was applied to identify conserved motifs of the AhHsfs full-length proteins.

### 2.4 Chromosomal localization and duplication event analysis

The chromosomal location image of peanut *Hsf* genes was generated by TBtools (Chen et al., 2020), according to the data obtained from the peanut genome database. The tandem and segmental duplications were identified by using MCScanX program (Wang et al., 2012) and the results were visualized by Circos (Krzywinski et al., 2009). The syntenic analysis of the orthologous genes obtained from peanut and other three plant species were investigated by TBtools (Chen et al., 2020). Subsequently, the synonymous substitution (Ks) and non-synonymous substitution (Ka) rates were calculated using DnaSP 5.0 software (Librado and Rozas, 2009).

### 2.5 Growth and stress treatments of peanut plants

The peanut cultivar HY9312 plants were used to analyze the expression of *AhHsf* genes in this study. The peanut seeds were germinated on MS medium in a light incubator at 25 °C for two weeks. The seedlings were then transferred to 20% PEG 6000 and 200 mM NaCl for drought and salt stresses, respectively. After treatments of 0, 6, 24, and 48 h, the leaves were harvested for RNA extraction and qRT-PCR analysis. The samples were frozen in liquid nitrogen immediately and then transferred to -80 °C for RNA extraction. Three biological replicates were used for each sample.

### 2.6 RNA extraction and qRT-PCR analysis

Total RNA was extracted by Trizol Reagent according to the manufacturer’s instructions. RNA quality and concentration were tested by using a Nanodrop 2000 spectrophotometer (Thermo Fisher Scientific, Wilmington, DE, United States). The synthesis of fist-strand cDNA by using the PrimeScript ™ RT reagent Kit (TaKaRa, Shiga, Japan) with Oligo (dT) primers. The qRT-PCR was performed on an ABI7500 Real-Time PCR System (Applied Biosystems, Foster City, CA, United States) with 2 µL template cDNA. The peanut *Actin11* gene was adopted as the internal control (Li et al., 2021). The relative primer sequences were listed in **Supplementary Table 1**. All reactions were performed with three biological replications and the resulting data were analyzed by using the 2 ^−ΔΔCT^ method (Livak and Schmittgen, 2001).

### 2.7 Subcellular localization

The coding regions (without stop codon) of *AhHsf20* were amplified from the cDNA of peanut leaves and inserted into the pCHF3-cGFP vector by Infusion (Invitrogen), generating the *AhHsf20-GFP* fusion fragment under control of the CaMV-35S promoter. The *35S::AhHsf20-GFP* plasmid was introduced into *Agrobacterium* competent cell GV3101 for transient expression in the leaf of *Nicotiana benthamiana*. After growth of three days in light chamber, these leaves were subjected to 4,6-diamidino-2-phenylindole (DAPI) staining to confirm the position of the nucleus. The fluorescence signals were monitored by using the confocal microscope (TCS-SP8 Leica, Wetzlar, Germany), as previously reported (Li et al., 2019).

### 2.8 Overexpression analysis

The coding sequence of *AhHsf20* was inserted into the *Sac*I-digested pCHF3 vector by Infusion (Clontech) to produce *35S::AhHsf20*, which was then transformed into *Agrobacterium tumefaciens* GV3101 and used to transform *Arabidopsis* Col-0 plants by the floral dip method (Zhang et al., 2006). T3 homozygous seedlings were used for further analyses. The sterilized wild-type and transgenic *Arabidopsis* seeds were evenly sown on 1/2 MS media. The seeds were stratified at 4 °C in darkness for two days, and then transferred to culture room (23 °C, continuous light). Wild-type and transgenic *Arabidopsis* seedlings growing normally for seven days were transferred to 1/2 MS media containing 100 and 150 mM NaCl. The primary root lengths were measured after seven days of upright growth in 1/2 MS media. Three biological replicates.

### 2.9 Statistical analysis

The GraphPad Prism 8 (*t* test) was used to analyze significant differences (*P* < 0.05, *P* < 0.01, *P* < 0.001). All data were obtained from three replicates.

## 3 Results

### 3.1 Identification of *Hsf* genes in peanut

To identify *Hsf* genes in peanut, the BLASTP search was performed using the Pfam Hsf-type DBD domain (PF00447) as query. After deleting redundant genes, we identified a total of 46 Hsf family proteins in peanut, designating the newly identified peanut *Hsf* genes *AhHsf1* to *AhHsf46* according to their position on chromosome. The Chr5, Chr13, and Chr15 contained the largest number of *AhHsf* genes, with six genes. There was followed by Chr3, which had five *AhHsf* genes. The Chr6 and Chr16 contained four *AhHsf* genes, respectively. The Chr8, Chr9, Chr17, and Chr19 contained two *AhHsf* genes, respectively. Additionally, Chr1, Chr2, Chr7, Chr10, Chr11, Chr12, and Chr20 contained only one *AhHsf* gene (**Supplementary Table 2**). The lengths of the predicted AhHsf proteins ranged from 207 amino acids (AhHsf29) to 599 amino acids (AhHsf44). The molecular weights (Mw) of Hsf proteins ranged from 23.6 (AhHsf29) to 66.7 (AhHsf44) kDa, and the theoretical isoelectric points (pI) ranged from 4.82 (AhHsf8 and AhHsf32) to 8.81 (AhHsf4) (**Supplementary Table 2**).

### 3.2 Phylogenetic analysis and classification of peanut *Hsf* genes

**Figure 1:**
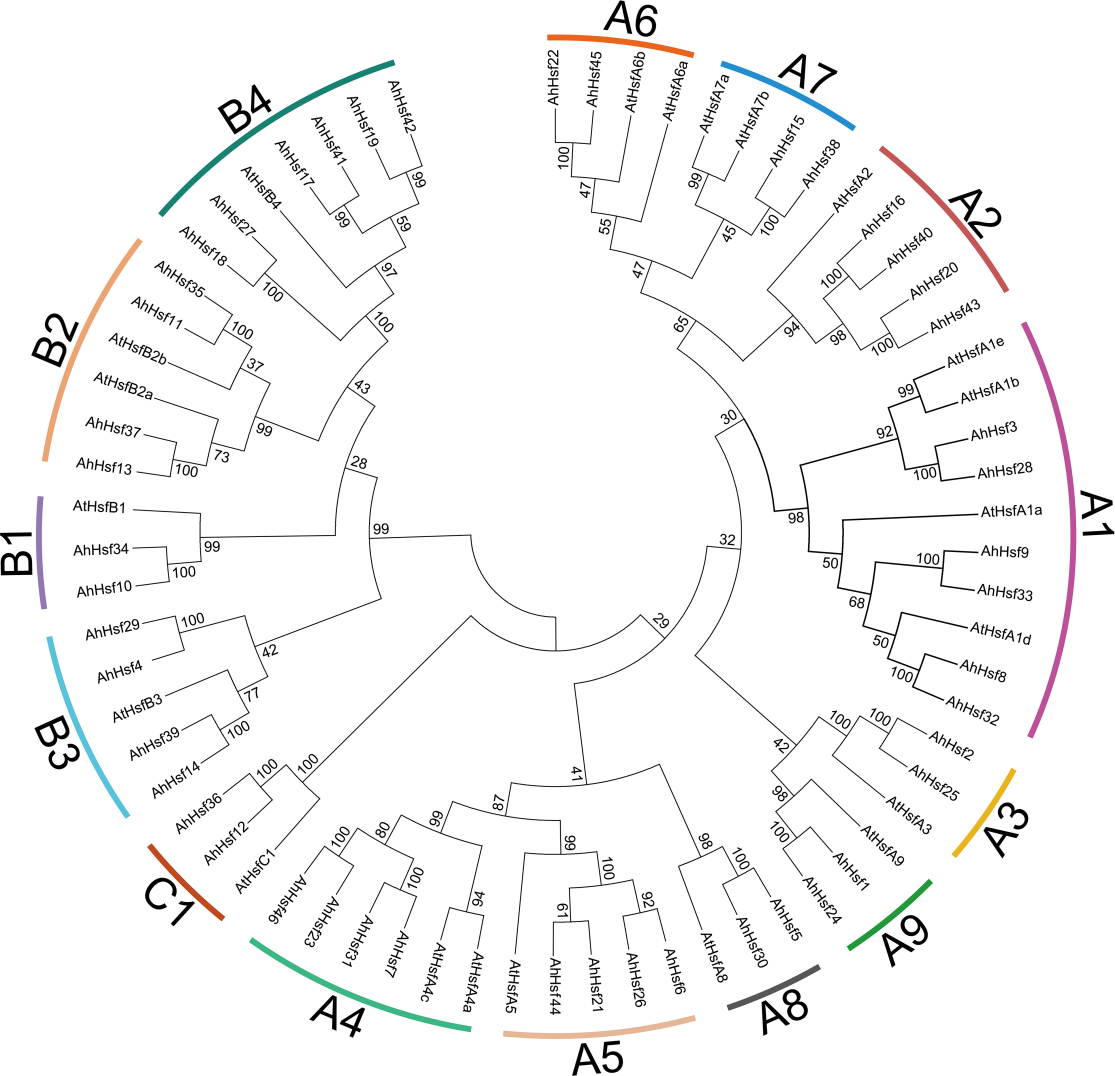
Phylogenetic analysis of peanut Hsf family members. The phylogenetic tree was generated from the alignment of peanut and *Arabidopsis* Hsf proteins with 1000 bootstrap replicates using the neighbor-joining (NJ) method. The peanut Hsf members together with their *Arabidopsis* homologs were classified into 14 subgroups.

In order to further study the evolutionary relationship among the peanut Hsf proteins, we constructed a neighbor-joining phylogenetic tree from alignments of 46 peanut Hsf proteins and 21 *Arabidopsis* Hsf proteins (**Figure 1**). The results showed that all the Hsf proteins from peanut and *Arabidopsis* were divided into three groups: A, B, and C. Furthermore, 28 AhHsfs in group A were divided into nine subgroups comprising A1-A9; 16 AhHsfs in group B were divided into four subgroups comprising B1, B2, B3, and B4; and 2 AhHsfs in group C were divided into C1 subgroup. The A1 and B4 subgroups contained the largest number of AhHsf members with six members. There were followed by subgroup A2, A4, A5, B2, and B3 subgroups, all of which had four members of the AhHsf family. Then subgroup A3, A6, A7, A8, A9, B1, and C1 subgroups all contained two members of the AhHsf family (**Figure 1**).

### 3.3 Conserved domains analysis of peanut Hsf proteins

We identified five typical conserved domains in peanut Hsf proteins, including DBD, OD, NLS, AHA, and NES domains (**Supplementary Table 3**). All of the AhHsf proteins possessed a highly conserved section, DBD domain, which contained three *α*-helices and four *β*-sheets (*α1*-*β1*-*β2*-*α2*-*α3*-*β3*-*β4*) in N-terminal region (**Supplementary Figure 1A**). Adjacent to the DBD domain is the HR-A/B domain, which is characterized by a coiled-coil structure in C-terminal region of the AhHsf proteins. We found that group A and group C Hsfs have different amounts of amino acid residues are respectively inserted between the HR-A and HR-B, whereas it is not found in the group B Hsfs (**Supplementary Figure 1B**). We also found that most of AhHsf proteins have NLS and NES domain, which are essential for maintaining the changes dynamically of Hsf proteins between nucleus and cytoplasm (Scharf et al., 1998; Heerklotz et al., 2001). 32 AhHsf proteins have NLS domain and NES was detected in 16 AhHsf members. Notably, the AHA motifs exist only in class A Hsfs. Among of them, seven AhHsf members (AhHsf8, AhHsf9, AhHsf23, AhHsf25, AhHsf32, AhHsf33, and AhHsf46) contained two AHA motif (**Supplementary Table 3** **).**

### 3.4 Gene structure and motif composition

**Figure 2:**
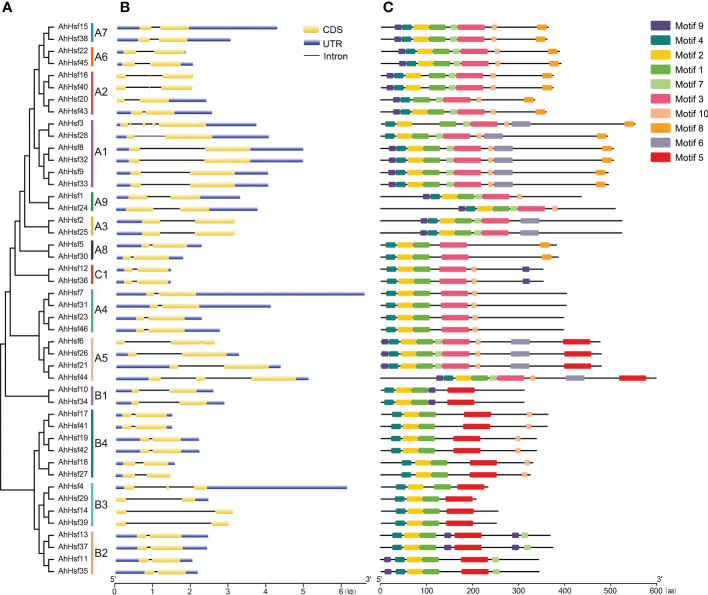
The motif and gene structure organizations of AhHsf members. **(A)** The evolutionary tree peanut Hsf family members. **(B)** Gene structure of *AhHsf* genes. **(C)** Protein motif of peanut Hsf members.

We analyzed the gene structure of all the *AhHsf* genes by GSDS, including introns and exons. The results showed that all *AhHsf* genes contained introns, but the number and length of introns were different (**Figure 2B**). In group A, there were four introns in *AhHsf3* gene, two introns in four *AhHsf* genes (*AhHsf6*, *AhHsf16*, *AhHsf40*, and *AhHsf44*), only one intron in others. In group B and group C, all *AhHsf* genes contained one intron. *AhHsf* genes had similar gene structures in the same subgroup.

We used MEME to analyze the motif composition of AhHsf proteins (**Figure 2C**). A total of 10 motifs (named motif 1-10), ranging from 6 to 50 amino acids, were predicted based on the feature of AhHsf protein sequences, and all sequences were listed in **Supplementary Figure 2**. Motif 1, motif 2, and motif 4 correspond to the DBD. Motif 3 and motif 5 correspond to the OD. Motif 10 correspond to the NLS. Motif 8 correspond to the NES.

### 3.5 Syntenic analysis

**Figure 3:**
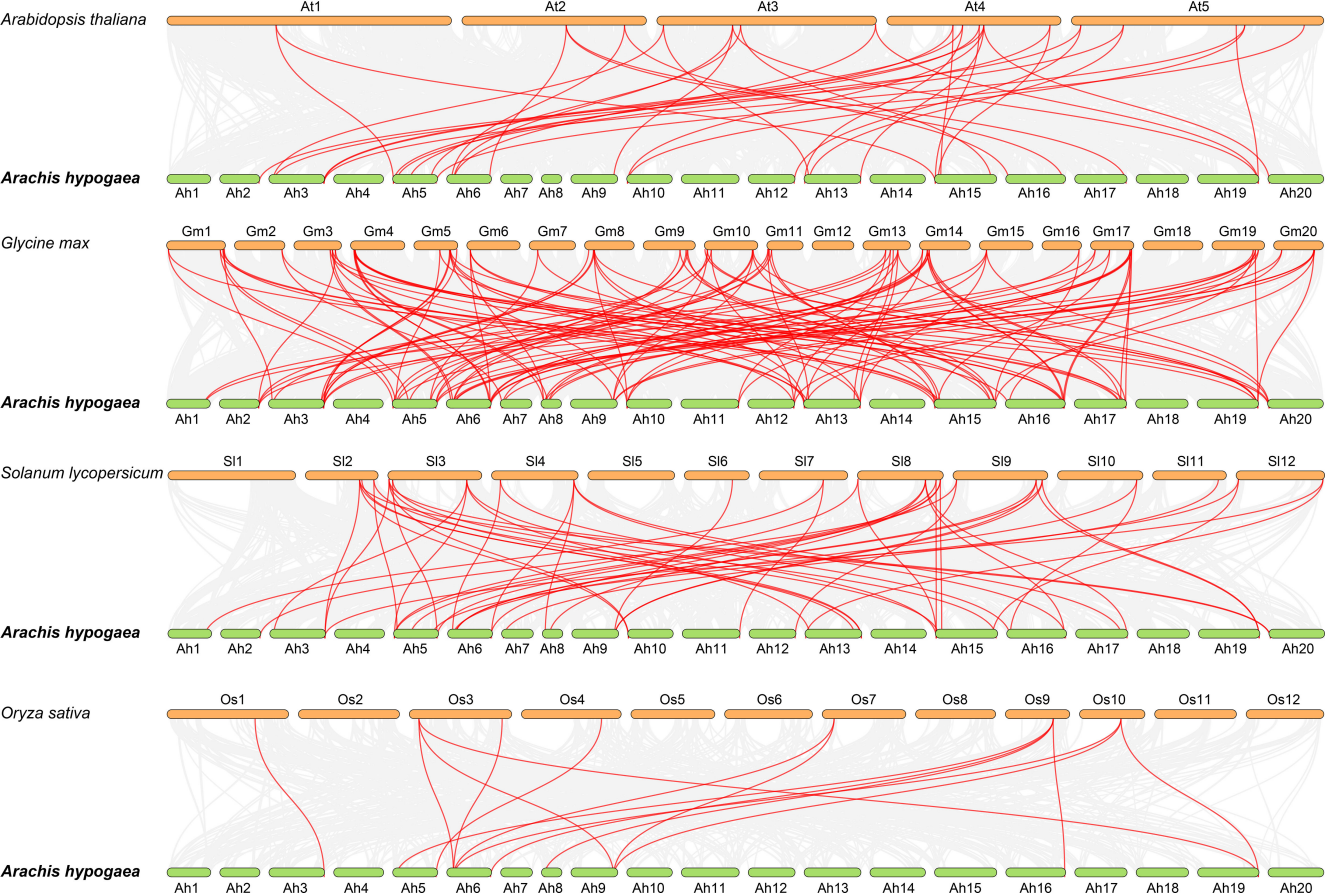
Synteny analysis of *Hsf* genes between peanut and four other plant species. The gray line in the background represented the collinear blocks between peanut and four other plant species, while the red line exhibited the syntenic *Hsf* gene pairs.

To further understand about the phylogeny of the peanut Hsf family members, syntenic analysis was performed between the *Hsf* genes of peanut and the *Hsf* genes of the other four plant species, including *Arabidopsis*, soybean (*Glycine max*), tomato (*Solanum lycopersicum*), and rice (*Oryza sativa*) (**Figure 3**). The results revealed that 39, 38, 27, and eight *AhHsf* genes were synchronized with *Hsf* genes in soybean, tomato, *Arabidopsis*, and rice, respectively. The number of collinear pairs between peanut and other four plant species (soybean, tomato, *Arabidopsis*, and rice), were 121, 52, 36, and 14, respectively, suggesting that the genetic relationship between peanut *Hsf* genes and soybean *Hsf* genes was close. Meanwhile, we found that the *AhHsf45* of peanut was associated with six, three, two, and two *Hsf* genes in soybean, *Arabidopsis*, tomato, and rice, respectively, suggesting that *AhHsf45* may play an important role during the evolution in peanut. Notably, a total of six *Hsf* genes formed collinear pairs with *Hsf* genes from all of other four plants, suggesting that these *Hsf* genes may have existed before the divergence of these plant species. The detailed information of syntenic gene pairs is provided in **Supplementary Table 4**.

### 3.6 Chromosomal distribution and duplication events

**Figure 4:**
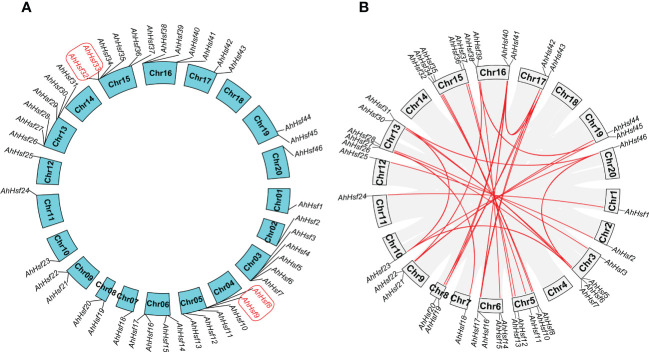
Chromosomal distribution and duplication events. **(A)** In peanut, 46 *AhHsf* genes were mapped on 17 peanut chromosomes. The tandem duplication pairs were featured by the red color. **(B)** The 35 putative segmental duplication pairs of *AhHsf* genes were investigated using MCScanX and linked by the colored lines, respectively. The gray lines indicate all putative segmental duplication pairs in the peanut genome, while the *AhHsf* segmental duplication pairs were linked by the red line.

In this study, 46 *AhHsf* genes were mapped on 17 peanut chromosomes, and each of which contains different number of the *AhHsf* genes (**Figure 4A**). The most peanut *Hsf* genes (six) were found on Ah5, Ah13, and Ah15, while Ah1, Ah2, Ah7, Ah10, Ah11, Ah12, and Ah20 only have one peanut *Hsf* gene. It has been reported that when a chromosome region within 200 kb possesses two or more genes of the same family, these genes are defined to be a gene cluster and genes sharing an identity of more than 70% in a cluster are considered to be tandem duplication genes (Holub, 2001). Homology analysis showed that there were two tandem duplication pairs (*AhHsf8*/*AhHsf9* and *AhHsf32*/*AhHsf33*) in peanut *Hsf* gene family (**Figure 4A**).

We used the MCScanX to analyze segmental duplication or whole genome duplication of the *AhHsf* genes. In a total, 35 segmental duplication pairs were identified in 42 *AhHsf* genes (**Figure 4B**). 23 pairs of segmental duplication occurred in group A *Hsf* genes. 11 pairs of segmental duplication occurred in group B *Hsf* genes. However, only one pair of segmental duplication (*AhHsf12*/*AhHsf36*) occurred in group C *Hsf* genes. These results suggested that the generation of some *Hsf* genes may be due to gene duplication events. All of the tandem and segmental duplication genes were listed in **Supplementary Table 5**.

Ka/Ks refers to the ratio between non-synonymous and synonymous substitutions, which can estimate whether the selective pressure acts on the protein-coding gene. There are three selection roles in evolutionary analysis, including the positive selection, neutral selection, and purifying selection. All Ka/Ks ratios of the 35 segmental and two tandem duplication pairs were less than one, suggesting that these *Hsf* genes may have undergone purifying selective pressure in process of evolution (**Supplementary Table 5**).

### 3.7 Promoter analysis of peanut *Hsf* genes

**Figure 5:**
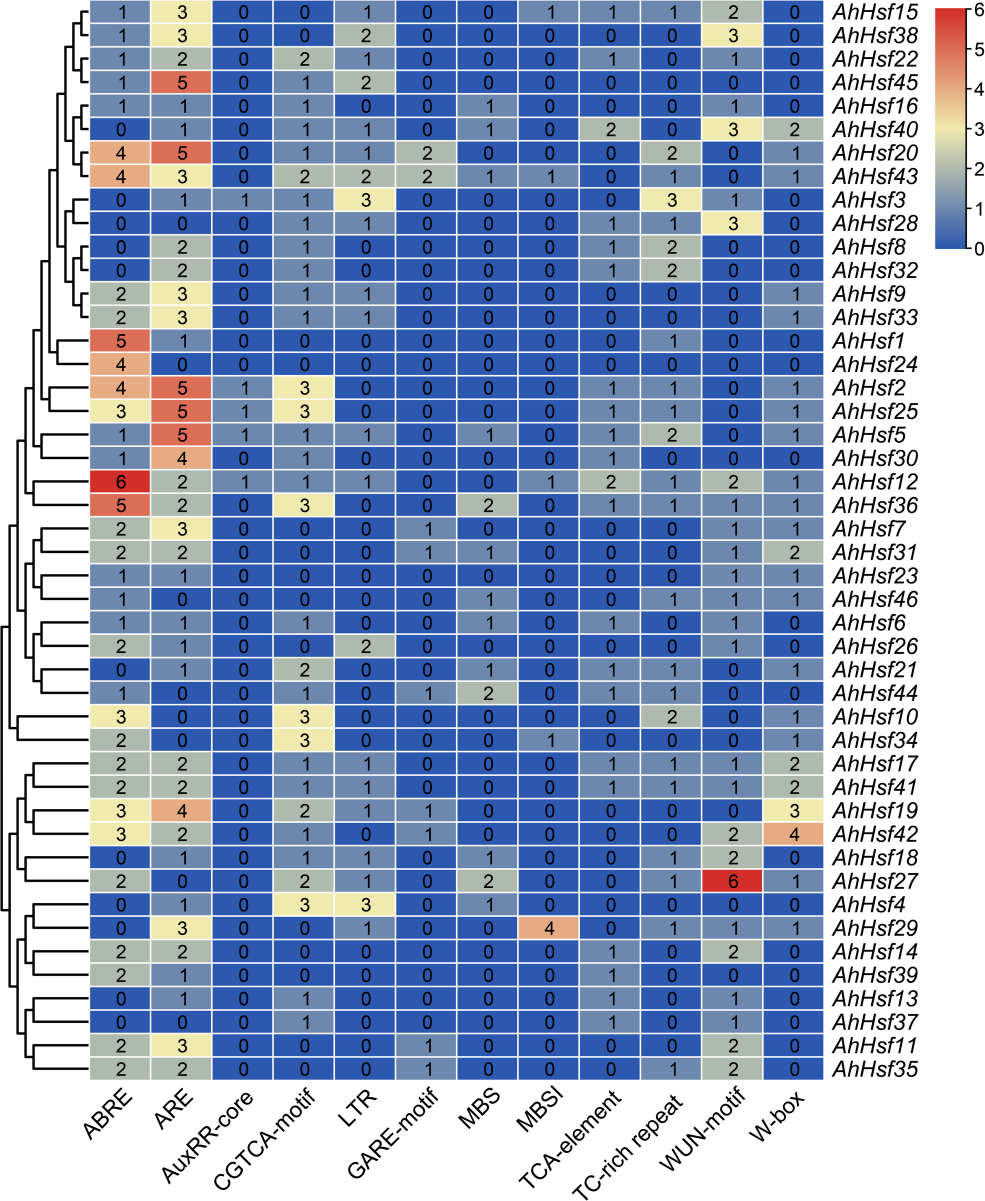
Regulatory elements in the promoter regions of *AhHsf* genes.

In order to study the potential function of *AhHsf* genes in stress responses, plantCARE was used to predict the *cis*-elements in promoter regions (**Figure 5**). Four hormone-responsive elements were identified in most *AhHsf* gene promoters, including ABRE, GARE, TCA-element, and CGTCA-motif, which regulate the plant responses to ABA, gibberellin, salicylic acid (SA), and methyl jasmonate (MeJA), respectively. The result suggested that most *AhHsf* genes may be involved in ABA-mediated and MeJA-mediated stress responses. Notably, the AuxRR-core element of regulating the plant responses to auxin was only detected in promoter regions of certain *AhHsf* genes, including *AhHsf2*, *AhHsf3*, *AhHsf5*, *AhHsf12*, and *AhHsf25*. Six stress-responsive elements including anaerobic induction element (ARE), wound-responsive element (WUN-motif), stress-responsive element (TC-rich repeats), WRKY binding site (W-box), MYB binding site (MBS), and low-temperature-responsive element (LTR) were also detected in the promoter regions. These *AhHsf* genes may be involved in responses to different stress conditions. Furthermore, a *cis*-element related to flavonoid biosynthetic (MBSI) was found in promoter regions of five *AhHsf* genes.

### 3.8 Expression patterns of peanut *Hsf* genes in different tissues

**Figure 6:**
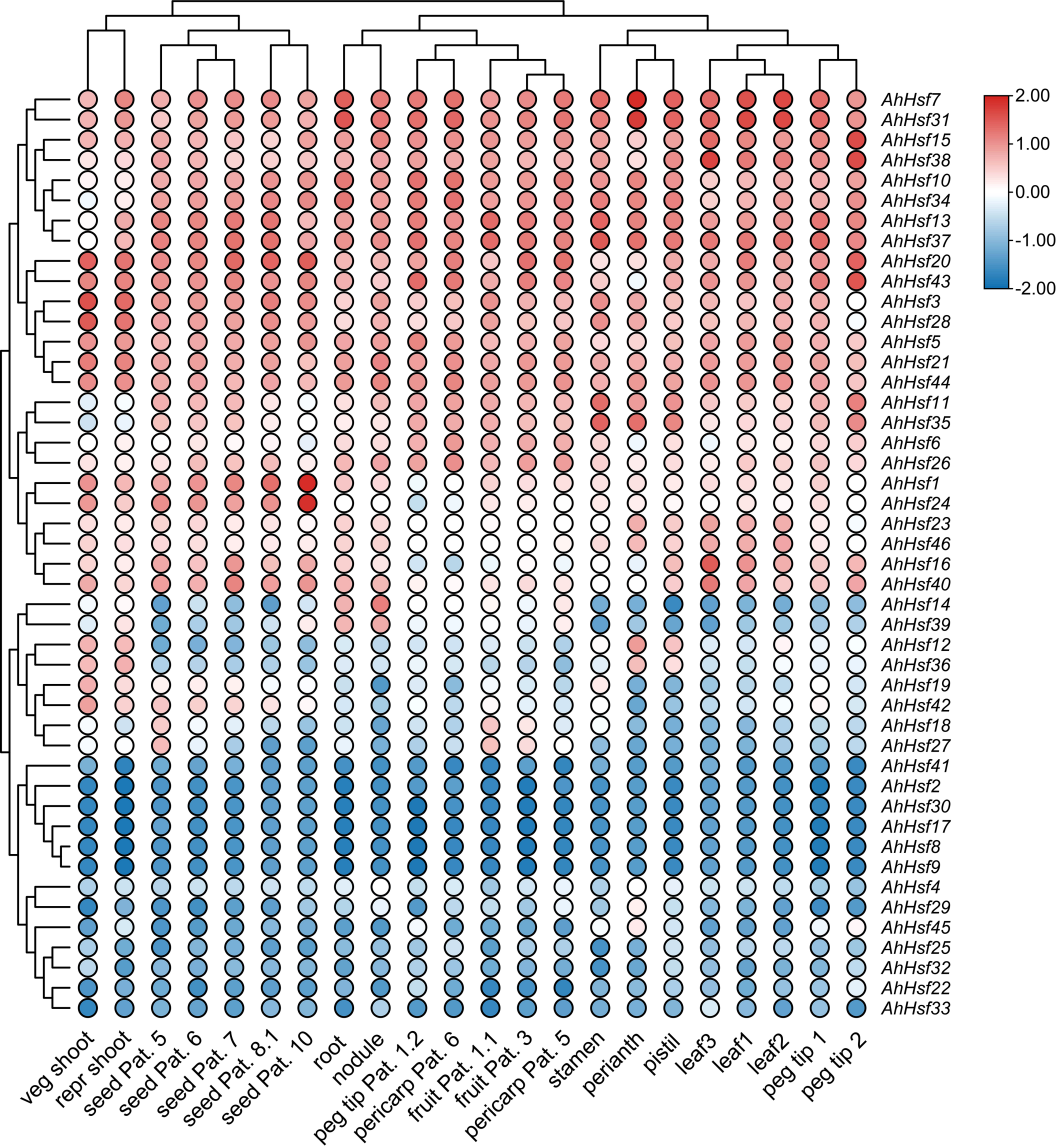
The expression patterns of *AhHsf* genes in 22 tissues. The FPKM values of each *AhHsf* gene were normalized and clustered using TBtools.

To determine the expression patterns of the *AhHsf* genes in different tissues, the corresponding transcriptome data with 22 tissues were accessed in the Peanut Base. It was found that the expressions of the *AhHsf* genes were significant differences in different tissues. As shown in **Figure 6**, *AhHsf5* from group A was highly expressed in the all-tested tissues, whereas the expression of its homologue *AhHsf30* was barely detectable. *AhHsf1* and *AhHsf24* were highly expressed in seed Pat. The expression levels of *AhHsf23* and *AhHsf46* in leaves were higher than other tissues. In group B, *AhHsf14* and *AhHsf39* were highly expressed in root and nodule. However, the expression of these two genes were lower in other tissues. Additionally, in group C, the expression levels of *AhHsf12* and *AhHsf36* were similar in the all-tested tissues. The various expression patterns suggested that *AhHsf* genes have different functions in peanut growth and development.

### 3.9 Expression of peanut *Hsf* genes under drought and salt stresses

**Figure 7:**
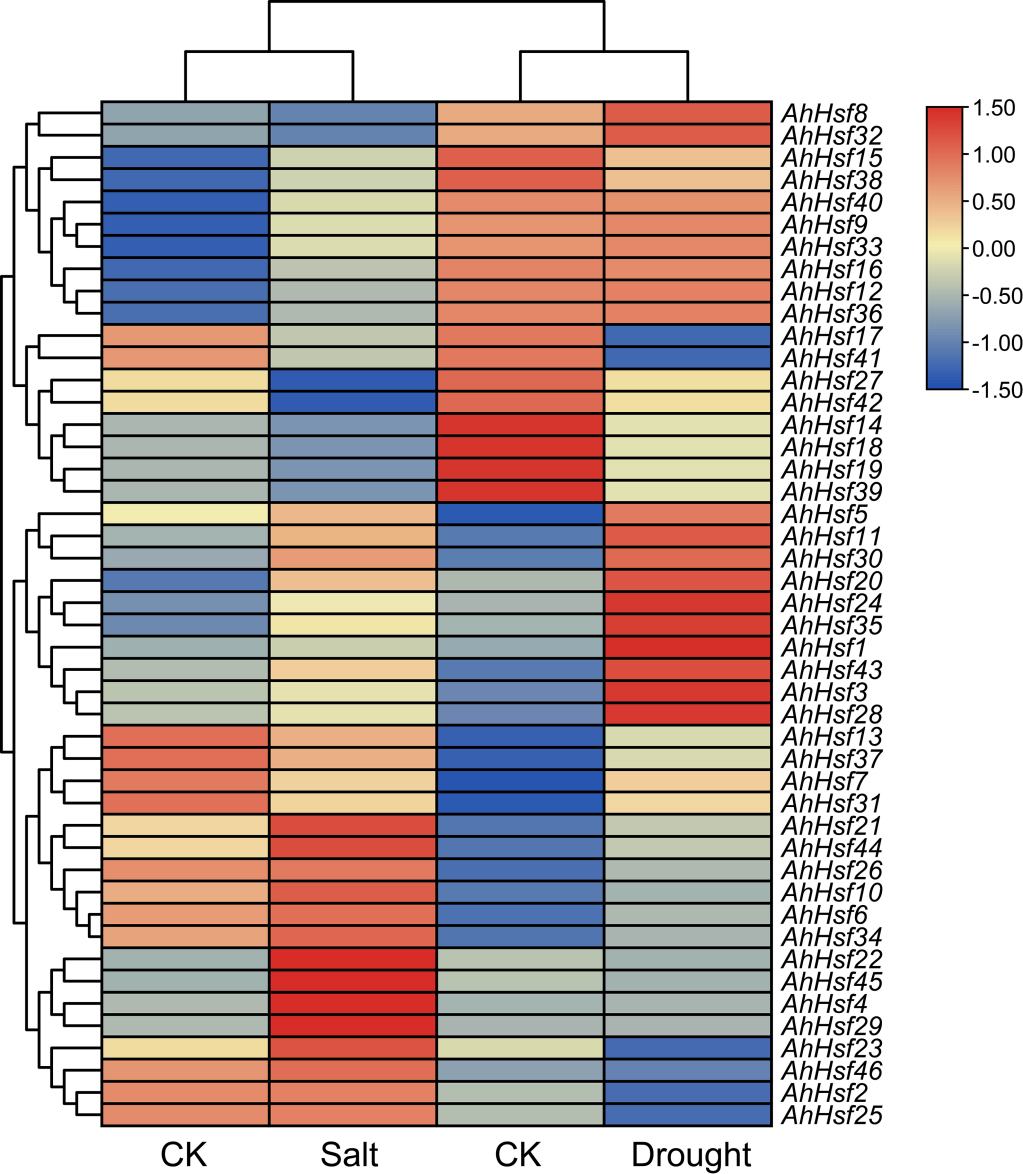
Heatmap of *AhHsf* genes under drought and salt stresses. The FPKM values of each *AhHsf* gene were normalized and clustered using TBtools.

To reveal peanut *Hsf* genes expression patterns under abiotic stresses, we utilized transcriptome data after drought and salt stresses from NCBI (Zhao et al., 2018; Zhang et al., 2020). As shown in **Figure 7**, most peanut *Hsf* genes exhibited several different expression patterns. Some genes, such as *AhHsf11*, *AhHsf24*, and *AhHsf35*, were upregulated under drought and salt stresses. Several peanut *Hsf* genes showed conflicting expression patterns under the two stress conditions. For instance, *AhHsf1* was insensitive to salt stress, whereas it was significantly induced by drought stress. Additionally, some genes did not show significant expression changes after drought and salt stress treatments, such as *AhHsf46*. The various expression patterns reflected the different roles of peanut *Hsf* genes in abiotic stress-response pathways.

### 3.10 Validation of expression patterns by qRT-PCR

**Figure 8:**
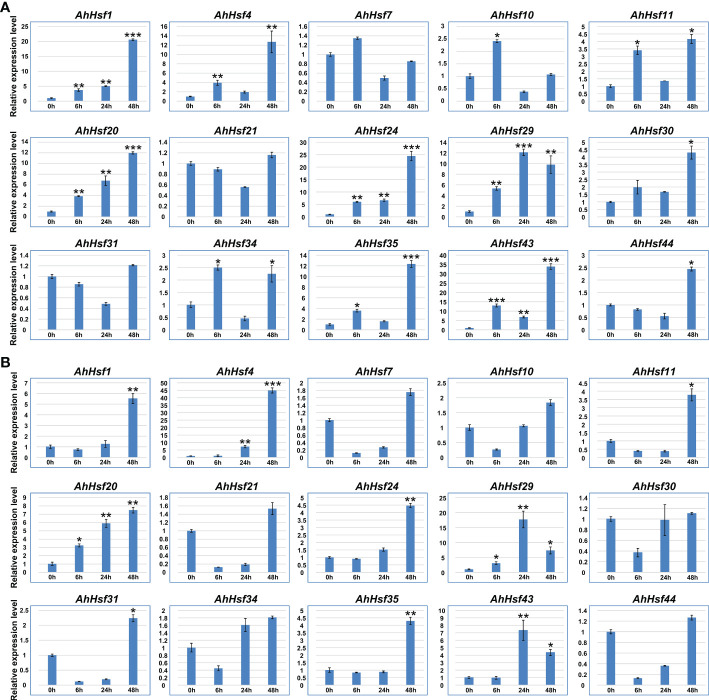
The expression patterns of *AhHsf* genes under drought and salt conditions. **(A)** The expression pattern of selected *AhHsf* genes in response to drought stress treatments, which was calculated as folds relative to the untreated control. **(B)** The expression pattern of selected *AhHsf* genes in response to salt stress treatments, which was calculated as folds relative to the control. The data were means ± SD of three biological repeats. *p < 0.05, **p < 0.01, ***p < 0.001 (*t*-tests).

qRT-PCR was used to verify the expression pattern of peanut *Hsf* genes in response to abiotic stresses, including drought and salt. According to the results, we found that several *AhHsf* genes were induced by drought and salt stresses (**Figure 8**). For example, the expression levels of *AhHsf1*, *AhHsf20*, and *AhHsf24* were continually up-regulated under drought stress, *AhHsf4* and *AhHsf24* were continually up-regulated under salt stress. Moreover, expression levels of *AhHsf1*/*4*/*20*/*24*/*29*/*35*/*43* were significantly increased (>10-fold) in drought stress condition. *AhHsf4*, *AhHsf20*, and *AhHsf29* were significantly increased (>7-fold) in salt stress condition. Notably, seven *AhHsf* genes, including *AhHsf1*, *AhHsf4*, *AhHsf20*, *AhHsf24*, *AhHsf29*, *AhHsf35*, and *AhHsf43*, were induced under both drought and salt stresses. These results suggested that peanut *Hsf* genes might be involved in response to drought and salt stresses.

### 3.11 Subcellular localization of AhHsf20

**Figure 9:**
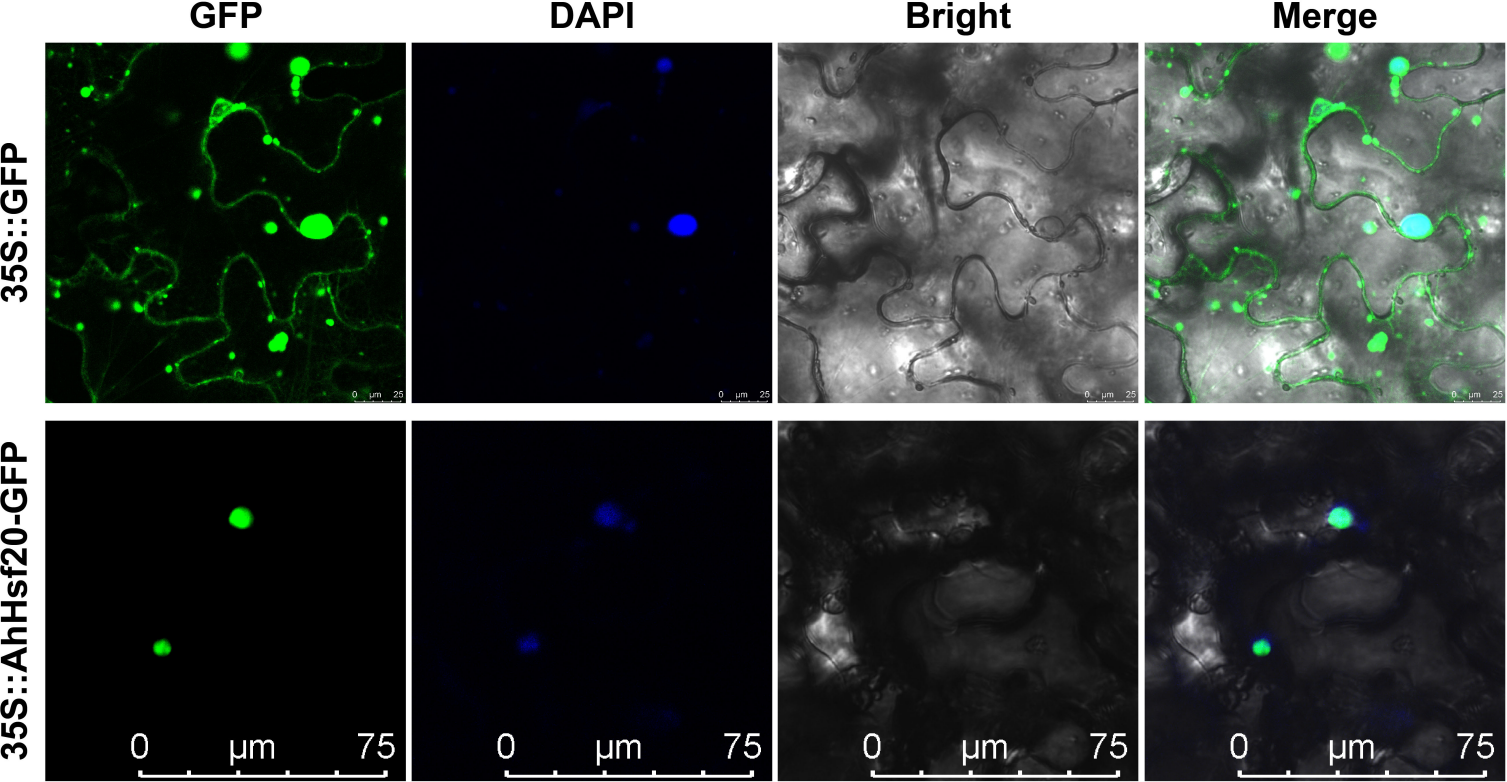
The subcellular localization of AhHsf20 in tobacco epidermal cells. The *AhHsf20-GFP* fusion construct and *GFP* gene driven by the CaMV-35S promoter were transiently expressed in tobacco, respectively. DAPI (dye 4,6-diamidino-2-phenylindole) staining indicted the nucleus.

In order to further explore the potential function of the *AhHsf* genes. The stress-induced *AhHsf20* gene was selected for subcellular localization analysis (**Figure 9**). The coding region of *AhHsf20* without the stop codon was fused to the GFP reporter gene under control of the CaMV35S promoter. The *Agrobacterium* cultures with the recombinant construct were used to inject the tobacco leaf epidermal cells, using 35S::GFP vector as the control. Subsequently, the signal of GFP protein was observed by confocal microscopy. It can be seen from **Figure 9** that AhHsf20 fusion protein was specifically located in the nucleus, whereas the control group was uniformly distributed throughout the whole cell. These results showed that AhHsf20 protein was located in nucleus.

### 3.12 Interaction networks of AhHsf in peanut

To explore the underlying mechanisms of action of AhHsf members, interaction networks of AhHsfs in peanut were predict using STRING online tool (https://cn.string-db.org/). We found that protein-protein interaction occurred between 18 peanut Hsf members (**Supplementary Figure 3**). There were four hub nodes in the AhHsf protein interaction network, including AhHsf13, AhHsf10, AhHsf2, and AhHsf18. Among them, AhHsf13 was found to interact with ten other Hsf members, followed by AhHsf2 (4), AhHsf10 (3), AhHsf18 (3). The results showed that these interacting proteins might co-exert specific biological functions in peanut.

### 3.13 Overexpression of *AhHsf20* enhanced salt tolerance in *Arabidopsis*

**Figure 10:**
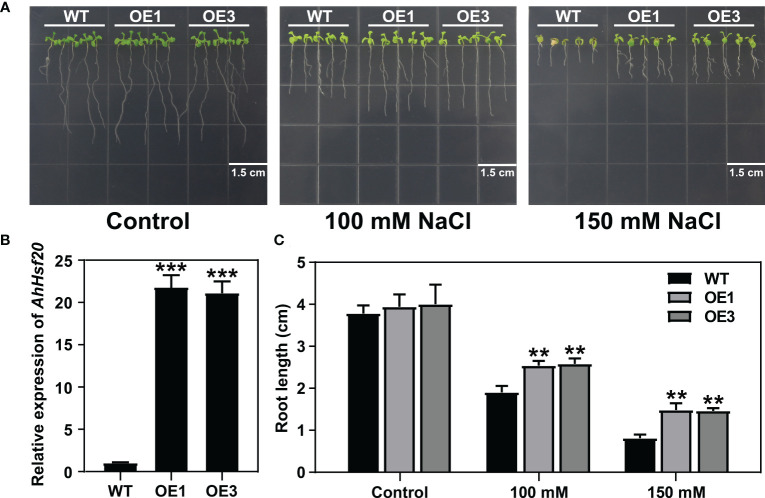
Effects of salt stress treatments on the root growth of the *AhHsf20* gene overexpressed in *Arabidopsis*. **(A)** Primary root lengths of the WT and *AhHsf20* overexpression lines under salt treatments in transgenic *Arabidopsis*. **(B)** The expression levels of *AhHsf20* gene in WT and two overexpression lines. The ratios of gene expression were calculated relative to the WT. **(C)** Quantification of the primary root lengths under normal condition and 100 and 150 mM NaCl treatments. The data were retrieved from three biological replicates. WT, wild type. Data are the mean ± SD of three biological repeats. **p < 0.01, ***p < 0.001 (*t* tests).

To further investigate the function of *AhHsf20* in response to abiotic stresses, we generated transgenic lines overexpressing *AhHsf20* under the control of the 35S promoter in *Arabidopsis* Col-0 plants (*AhHsf20-OE*). Two overexpression lines *AhHsf20-OE1* and *AhHsf20-OE3* were selected for further studies (**Figure 10B**). Compared with wild-type, *AhHsf20-OE1* and *AhHsf20-OE3* plants displayed a longer root phenotype under 100 and 150 mM NaCl (**Figures 10A, C**). The results revealed that the overexpression of *AhHsf20* enhanced the salt tolerance in transgenic *Arabidopsis*.

## 4 Discussion

It has been reported that *Hsf* genes are heat shock transcription factors and play important roles in response to abiotic stresses and plant development processes (von Koskull-Doring et al., 2007). Cultivated peanut (*Arachis hypogaea* L.) is not only an important economic crop but also an oil crop. Thus, identification and analysis *Hsf* genes in peanut will be great significance on various stress responses. Previous study found that 16 and 17 *Hsf* genes were identified from *Arachis duranensis* and *A. ipaensis*, respectively (Wang et al., 2017). However, the *Hsf* gene family in cultivated peanut has not been comprehensive analyses so far. There are only 21 *Hsf* genes in *Arabidopsis* (Guo et al., 2008). In the present study, a total of 46 *Hsf* genes were identified from peanut, probably due to peanut is an allopolytraploid. Consistent with the classification of *Hsf* genes in other plant species including *Arabidopsis*, rice (Guo et al., 2008), potato (Tang et al., 2016), and tomato (Yang et al., 2016), peanut *Hsf* genes were also divided into three groups: A, B, and C (**Figure 1**). There are 28 *Hsf* genes belong to group A (A1-A9), 16 in group B (B1-B4), and two in group C (C1).

Conserved motif analysis suggested that all of 46 AhHsf proteins contain two necessary domains (DBD and OD) and/or three specific domains (NLS, AHA, and NES). The transcriptional activation activities of Hsf from group A were worked by the AHA motif in the C-terminal region (Doring et al., 2000). In the peanut Hsf family, 27 of 28 AhHsfA members with AHA motif (**Supplementary Table 3**), suggesting that these AhHsf members may have self-transcriptional activation activity, whereas the AhHsf16 from subgroup A2 without AHA motif. It has been reported that HsfA members with no AHA motif might be activated by forming hetero-polymers with other group A Hsf (Scharf et al., 2012). Furthermore, the gene structures of peanut *Hsf* genes are highly conserved within the same subgroup (**Figure 2**), further supporting the result of evolutionary analysis.

Segmental duplication and tandem duplication events contribute to the expansion of the gene families in plant genomes (Vision et al., 2000; Cannon et al., 2004). In this study, we found that the expansion of the *AhHsf* genes in peanut is mainly caused by segmental duplication, not tandem duplication. A total of 35 segmental and two tandem duplication pairs were identified (**Figure 4**), with all the Ka/Ks ratios of these duplication pairs were less than one (**Supplementary Table 5**), suggesting that these duplicated peanut *Hsf* genes might have undergone purifying selective pressure in process of peanut evolution. In addition, duplicated peanut *Hsf* genes belonged to the same subgroup, such as *AhHsf8*/*AhHsf9* in subgroup A1 and *AhHsf10*/*AhHsf34* in subgroup B1. Notably, *AhHsf10* and *AhHsf34*, as a segmental duplication pair, exhibited consistent organ/tissue expression patterns (**Figure 6**).

Most of the Hsf transcription factors have been reported to be involved in the regulation of *Arabidopsis* developmental processes. For example, AtHsfA9 from subgroup A9 was reported to be involved in regulation of seed development in *Arabidopsis* (Kotak et al., 2007). A segmental duplication pair *AhHsf1* and *AhHsf24* was clustered together with AtHsfA9 in subgroup A9, and these two genes both highly expressed in seeds (**Figure 6**), suggesting that *AhHsf1* and *AhHsf24* might also be involved in regulating seed development. In addition, AtHsfB2a was shown to play important role in regulating gametophyte development of *Arabidopsis* (Wunderlich et al., 2014). Its homolog *AhHsf13* showed high expression level in stamen (**Figures 3**, **6**), which suggested that AtHsfB2a and AhHsf13 might have similar biological functions.

Drought and salt stresses are the two major abiotic stresses adversely affecting the growth development and yields of crops (Suzuki et al., 2014). The plant cells will exhibit consistent changes in water loss under drought or salt stress. Osmotic adjusting can enhance water absorption to cope with salt and drought stresses, simultaneously (Munns et al., 2020; Ozturk et al., 2021). For example, under salt and drought stresses, a Chloride (Cl-)-tolerant species *Pugionium cornutum* that can accumulate Cl- and enhance osmotic adjustment capacity to improve growth (Cui et al., 2020). Notably, the accumulation of osmotic substances can be realized by altering relative gene expression levels. In tomato, transgenic *SlWRKY8* plants exhibit enhanced salt and drought tolerance, with higher osmotic substances like proline (Gao et al., 2020). It is worth noting that a number of Hsf members have been reported to be involved in response to plant environment stresses. In subgroup A1, *AtHsfA1b* has been reported to enhance drought resistance (Bechtold et al., 2013). Its peanut homologs *AhHsf3* and *AhHsf28* were found to be induced by drought stress (**Figures 3**, **7**), implying that these *AhHsf* genes might be involved in regulating drought stress response. Furthermore, *AhHsf3* and *AhHsf28* were predicted to have arisen from segmental duplication events (**Figure 4B**). In subgroup A6, AtHsfA6b was demonstrated to induce stress-responsible genes under salt or drought stresses via the ABA dependent signaling pathway (Huang et al., 2016). *AhHsf22* and *AhHsf45* were clustered together with *AtHsfA6b* in subgroup A6, and both were induced by salt stress (**Figure 7**). Furthermore, the promoter regions of *AhHsf22* and *AhHsf45* have one ABA-responsive element (ABRE) respectively (**Figure 5**), suggesting that these peanut *Hsf* genes might be involved in response to salt stress through the ABA signaling pathway. Notably, salt and drought stress treatments can significantly induce the expression of *AhHsf20* (**Figures 7**, **8**), implying that *AhHsf20* may be involved in salt- and drought-stress responses. Further experiments indicated that the overexpression of *AhHsf20* can enhance salt tolerance in transgenic *Arabidopsis* (**Figure 10**), supporting the potential roles of more AhHsf proteins in stress responses.

## 5 Conclusion

In this study, a systematic analysis of the peanut Hsf gene family was performed, and a total of 46 AhHsf members were identified. They were divided into three groups and 14 subgroups together with their *Arabidopsis* homologs. Our analyses of Hsf genes of peanut and *Arabidopsis* revealed their diversity in the number of members, evolutionary relationship, gene structure, chromosome locations, gene duplication, collinearity, tissue expression patterns and expression patterns in response to salt and drought stresses. The results indicate that the *AhHsf* genes may be important for regulating peanut responses to abiotic stresses and development. Notably, *AhHsf20* was induced by salt and drought stresses and was able to enhance salt tolerance in transgenic *Arabidopsis*. Overall, these results from this study can help us to further examine the specific functions of the *AhHsf* genes and provide some new genes resource for peanut stress resistance breeding.

## Data availability statement

The original contributions presented in the study are included in the article/**Supplementary Material**. Further inquiries can be directed to the corresponding authors.

## Author contributions

PC and SS conceived this research, designed the experiments. QW, Z, and CG conducted the research and drafted the manuscript. XZ, ZL, YM, QS, JW, CY, and CL assisted in data collection and analysis. All authors have read and approved the final version of the manuscript.

## Supplementary material

Supplementary Figure 1 Multiple sequence alignment of the DBD domains **(A)** and HR-A/B regions **(B)** of the Hsf protein family in peanut.

Supplementary Figure 2 The putative conserved motifs in AhHsf proteins.

Supplementary Figure 3 Interaction networks of AhHsf in peanut.

Supplementary Table 1 The qRT-PCR primers used in this study.

Supplementary Table 2 The detail information of identified peanut Hsf family members.

Supplementary Table 3 The functional domains and motifs of peanut Hsf members.

Supplementary Table 4 The syntenic pairs between peanut and other four plant species.

Supplementary Table 5 The detail information of segmental and tandem duplication gene pairs.
