# Supplementary material for: Hsf transcription factor gene family in peanut (Arachis hypogaea L.): genome-wide characterization and expression analysis under drought and salt stresses
Source: Front Plant Sci. 2023 Jul 5;14:1214732. doi: 10.3389/fpls.2023.1214732 (PMC10355374; doi:10.3389/fpls.2023.1214732)
Supplement: Supplementary Figure 1 — Multiple sequence alignment of the DBD domains (A) and HR-A/B regions (B) of the Hsf protein family in peanut. [file DataSheet_1.zip › Supplementary Materials/Supplementary Figure S1.pdf]

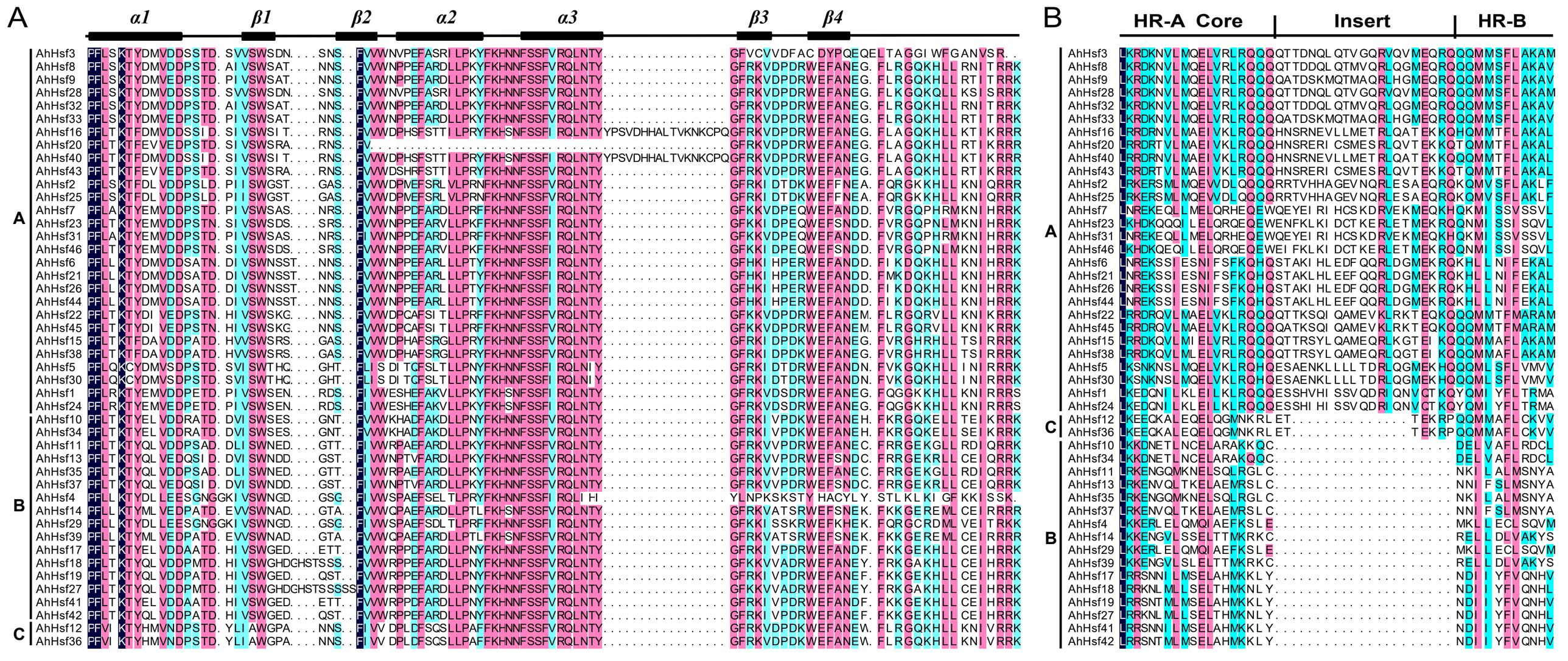
A
α1
β1
β2
α2
α3
β3
β4
AhHsf3
AhHsf8
AhHsf9
AhHsf28
AhHsf32
AhHsf33
AhHsf16
AhHsf20
AhHsf40
AhHsf43
AhHsf2
AhHsf25
AhHsf7
AhHsf23
AhHsf31
AhHsf46
AhHsf6
AhHsf21
AhHsf26
AhHsf44
AhHsf22
AhHsf45
AhHsf15
AhHsf38
AhHsf5
AhHsf30
AhHsf1
AhHsf24
AhHsf10
AhHsf34
AhHsf11
AhHsf13
AhHsf35
AhHsf37
AhHsf4
AhHsf14
AhHsf29
AhHsf39
AhHsf17
AhHsf18
AhHsf19
AhHsf27
AhHsf41
AhHsf42
AhHsf12
AhHsf36
A
B
C
B
HR-A Core
Insert
HR-B
AhHsf3
AhHsf8
AhHsf9
AhHsf28
AhHsf32
AhHsf33
AhHsf16
AhHsf20
AhHsf40
AhHsf43
AhHsf2
AhHsf25
AhHsf7
AhHsf23
AhHsf31
AhHsf46
AhHsf6
AhHsf21
AhHsf26
AhHsf44
AhHsf22
AhHsf45
AhHsf15
AhHsf38
AhHsf5
AhHsf30
AhHsf1
AhHsf24
AhHsf12
AhHsf36
AhHsf10
AhHsf34
AhHsf11
AhHsf13
AhHsf35
AhHsf37
AhHsf4
AhHsf14
AhHsf29
AhHsf39
AhHsf17
AhHsf18
AhHsf19
AhHsf27
AhHsf41
AhHsf42
A
C
B
