# Supplementary figures and images for: Hsf transcription factor gene family in peanut (Arachis hypogaea L.): genome-wide characterization and expression analysis under drought and salt stresses
Source: Front Plant Sci. 2023 Jul 5;14:1214732. doi: 10.3389/fpls.2023.1214732 (PMC10355374; doi:10.3389/fpls.2023.1214732)

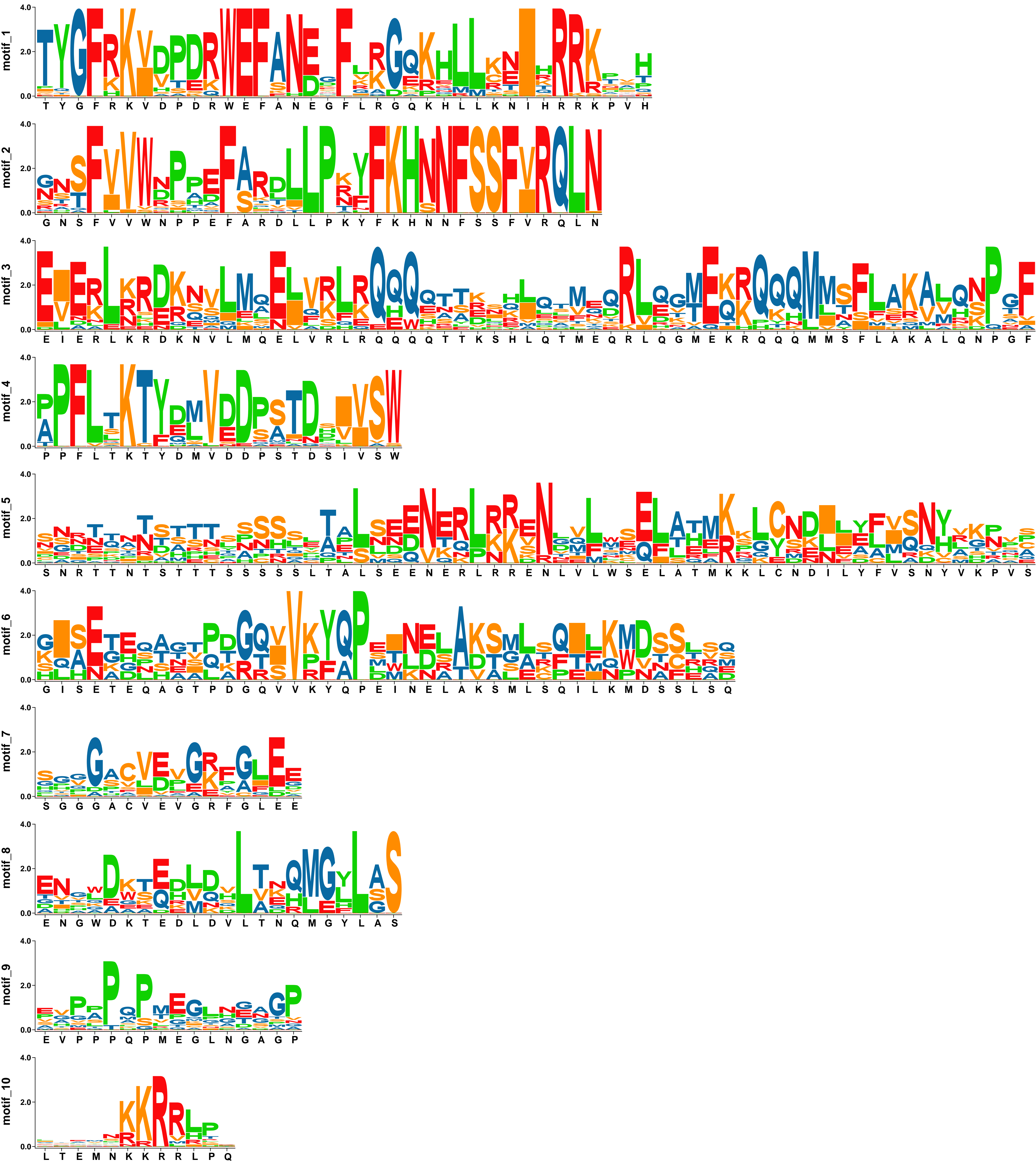

Supplement: Supplementary Figure 1 — Multiple sequence alignment of the DBD domains (A) and HR-A/B regions (B) of the Hsf protein family in peanut. [file DataSheet_1.zip › Supplementary Materials/Supplementary Figure S2.pdf]

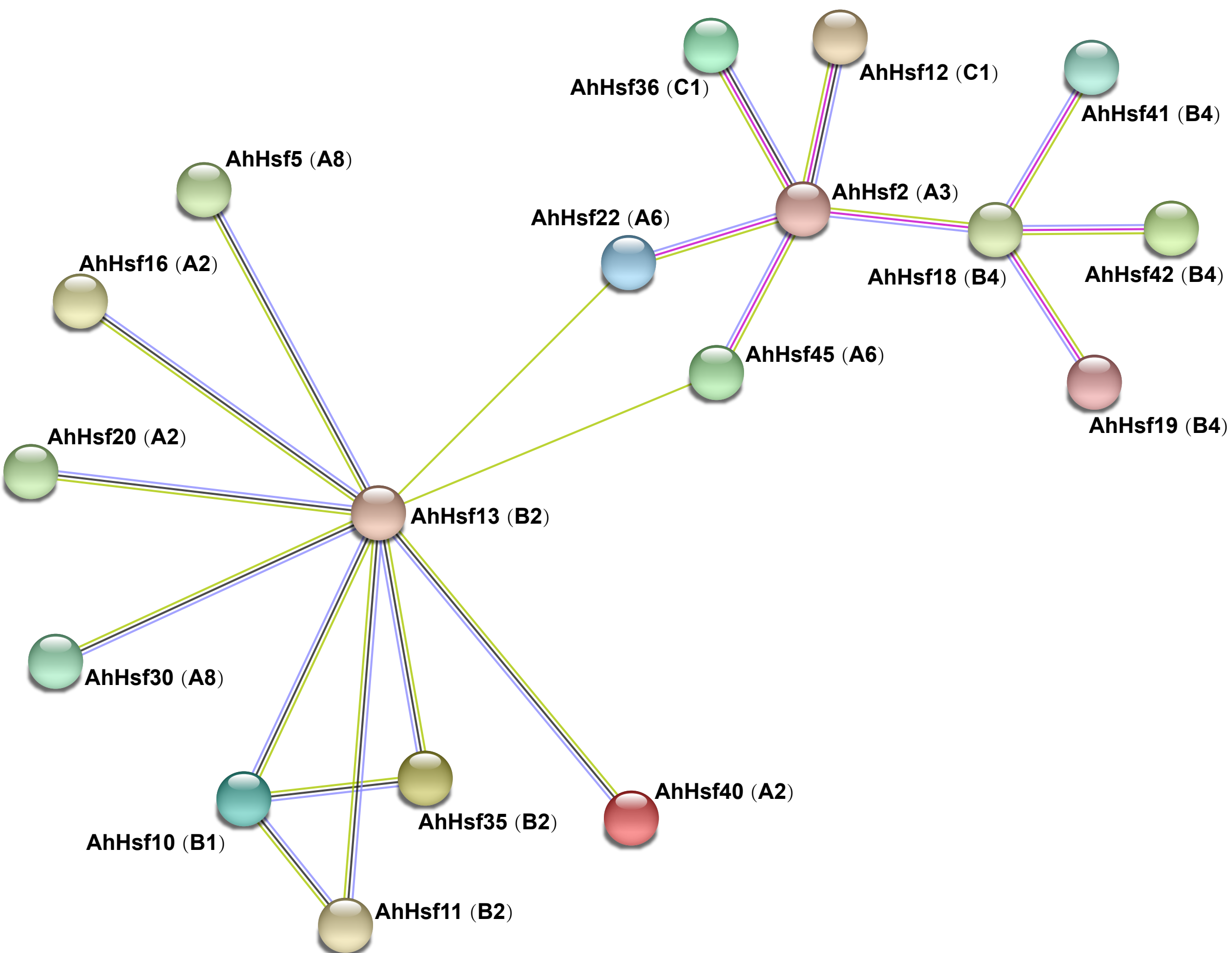

Supplement: Supplementary Figure 1 — Multiple sequence alignment of the DBD domains (A) and HR-A/B regions (B) of the Hsf protein family in peanut. [file DataSheet_1.zip › Supplementary Materials/Supplementary Figure S3.pdf]
